# Multiple Mutations in Heterogeneous Miltefosine-Resistant *Leishmania major* Population as Determined by Whole Genome Sequencing

**DOI:** 10.1371/journal.pntd.0001512

**Published:** 2012-02-14

**Authors:** Adriano C. Coelho, Sébastien Boisvert, Angana Mukherjee, Philippe Leprohon, Jacques Corbeil, Marc Ouellette

**Affiliations:** Centre de Recherche en Infectiologie, Université Laval, Québec, Canada; SBRI, United States of America

## Abstract

**Background:**

Miltefosine (MF) is the first oral compound used in the chemotherapy against leishmaniasis. Since the mechanism of action of this drug and the targets of MF in *Leishmania* are unclear, we generated in a step-by-step manner *Leishmania major* promastigote mutants highly resistant to MF. Two of the mutants were submitted to a short-read whole genome sequencing for identifying potential genes associated with MF resistance.

**Methods/Principal Findings:**

Analysis of the genome assemblies revealed several independent point mutations in a P-type ATPase involved in phospholipid translocation. Mutations in two other proteins—pyridoxal kinase and α-adaptin like protein—were also observed in independent mutants. The role of these proteins in the MF resistance was evaluated by gene transfection and gene disruption and both the P-type ATPase and pyridoxal kinase were implicated in MF susceptibility. The study also highlighted that resistance can be highly heterogeneous at the population level with individual clones derived from this population differing both in terms of genotypes but also susceptibility phenotypes.

**Conclusions/Significance:**

Whole genome sequencing was used to pinpoint known and new resistance markers associated with MF resistance in the protozoan parasite *Leishmania*. The study also demonstrated the polyclonal nature of a resistant population with individual cells with varying susceptibilities and genotypes.

## Author Summary

*Leishmania* spp. are parasitic protozoa responsible for a spectrum of diseases known as leishmaniasis. There are few drugs available for the treatment of these diseases, and miltefosine is the first oral drug used in treatment of visceral leishmaniasis, a form of the disease that can be lethal if not treated. In this study, we seek to understand the mechanism of action and identify targets of the drug by generating promastigote mutants highly resistant to miltefosine. Two independent mutants were submitted to short read whole genome sequencing. Genome analysis of these mutants has permitted us to identify point mutations in three genes (P-type ATPase, pyridoxal kinase and α-adaptin like protein) that were also present in other independent miltefosine resistant mutants. Some of the new genes identified here could be useful as potential markers for miltefosine resistance in *Leishmania*. Moreover, our approach has permitted us to highlight that resistance can be highly heterogeneous at the population level with individual clones derived from this population differing both in terms of genotypes but also susceptibility phenotypes. This may have practical applications while studying resistance.

## Introduction

*Leishmania* is a protozoan parasite responsible for a spectrum of diseases collectively known as leishmaniasis in tropical and subtropical areas of the world [1]. There is no effective vaccine for the prevention of this parasitic disease and its control relies on chemotherapy. The arsenal of available drugs is limited with most compounds being compromised by toxicity, cost, or resistance [2]. The alkyl-lysophospholipid analogue miltefosine (MF), a drug initially developed as an antitumoral compound, was the first effective oral drug against *Leishmania*. It has been successfully used in the last decade for the treatment of visceral leishmaniasis [3]–[5]. The exact mode of action of MF is not well understood, but it was shown to induce changes in the biosynthesis of phospholipids and the metabolism of alkyl-lipids in *Leishmania* [6], in addition to induce an apoptotic cell death [7], [8]. The uptake of MF and other alkyl-glycerophospholipids in *Leishmania* requires a P-type ATPase, named miltefosine transporter (MT), which is responsible for the translocation of phospholipids from the exoplasmic to the cytoplasmic leaflet of the plasma membrane of the parasite [9]. Based on findings in yeast [10], [11], the uptake of MF in *L. donovani* was further shown to require a protein named LdRos3, the β-subunit of the MT [12]. Differences in susceptibility to alkyl-lysophospholipids between *Leishmania* species [13], [14] has recently been associated with the low expression of this MF translocation machinery in species displaying a lack of intrinsic MF susceptibility [15].

Resistance to MF in *Leishmania* [12] and other organisms [16], [17] involved a decreased accumulation of the drug due to reduced uptake or increased efflux. In *L. donovani*, a drastic reduction (40-fold decrease) in the ability to internalize the drug was shown to be accompanied by a decreased content of unsaturated phospholipids in the plasma membrane of resistant parasites [18], [19]. The acquisition of inactivating mutations or the deletion of the *MT* gene in *L. donovani* [9], [20] was shown to drastically increase MF resistance both *in vitro* and *in vivo*. In addition, the overexpression of ABCG4 and ABCG6, two ABC proteins localized to the flagellar pocket and plasma membrane of the parasite, was associated with an increased resistance to several alkyl-lysophospholipid analogues in *Leishmania* [21]–[23]. Parasites overexpressing ABCG4 or ABCG6 had a reduced accumulation of fluorescent phospholipids and [^14^C]-MF, which suggested an enhanced outward translocation of the drug across the plasma membrane. A *L. tropica* mutant resistant to daunomycin harboring an amplification of the ABC protein ABCB4 (MDR1) was also shown to display cross-resistance to MF [24], although the absence of similar amplicons in MF-resistant *L. donovani* suggested that the amplification of ABCB4 (MDR1) is not a frequent mechanism of resistance to MF [25]. Finally, functional cloning experiments identified a hypothetical gene in *L. infantum* whose increased expression conferred resistance to MF and antimony [26].

The central role of MF in the control of leishmaniasis warranted further studies on its mode of action and the mechanisms involved in resistance. Indeed, the ease with which MF-resistant parasites are selected *in vitro* suggests that clinical failures resulting from MF-resistant isolates are likely. In this study, we sought to determine the mutational events involved in MF resistance on a whole genomic scale by sequencing the entire genome of two *L. major* mutants independently selected for high level MF resistance *in vitro*.

## Methods

### Cell lines, culture conditions and transfections

*L. major* Friedlin and *L. infantum* (MHOM/MA/67/ITMAP-263) wild-type promastigotes were grown at 25°C in SDM-79 medium supplemented with 10% heat inactivated fetal bovine serum and 10 µg/ml hemin. The *L. major* Friedlin MF80.1, MF80.2, MF80.3 and MF80.5 mutants were selected from a cloned parental population using a stepwise selection until they were resistant to 80–100 µM MF. MF was purchased from Cayman Chemical (Ann Harbor, USA). To obtain clones derivied from the mutants 5.3, 10.3, 15.3 40.3 and 80.3, parasites were spread on SDM-agar (1% Noble Agar, Nunc) plates in absence of MF.

Partial revertants were obtained by culturing the resistant lines in the absence of MF for 30 passages. The *L. infantum* MF200.3 and MF200.5 mutants selected for MF resistance had been generated previously [27]. Growth curves were obtained by measuring absorbance at 600 nm as previously described [28]. Gene transfection was performed by electroporation as reported previously [29]. Statistical significance was determined by Student's *t*-test. Significance was considered as P<0.05.

### DNA manipulations

Total DNA was isolated using DNAzol reagent (Invitrogen) as recommended by the manufacturer. For quantitative Southern blots, the genomic DNA was digested with appropriate restriction enzymes and migrated in 0.8% agarose gels. Southern blots, hybridizations and washes were performed following standard protocols [30]. Probes were obtained by amplifying the upstream or downstream regions of the gene studied. The pSP72αHYGα-MT construct was generated by the amplification of the MF transporter gene from *L. major* Friedlin (LmjF13.1350) with primers containing the restriction enzymes *Xba*I and *Hind*III (Table S1) and cloned in the respective plasmid. All the constructs were checked by sequencing with internal primers (Table S1). Similarly, the genes pyridoxal kinase (LmjF30.1250) and α-adaptin like protein (LmjF07.0050) were cloned in pSP72αNEOα or pSP72αHYGα[31] at *Xba*I and *Hind*III sites.

The pyridoxal kinase gene (LmjF30.1250) inactivation cassette was generated by overlap extension PCR [32], [33] using the primer A-PK-KO and primer B-PK-NEO-KO to amplify a region of 550-bp upstream of the gene; primer E-PK-NEO-KO and primer F-PK-KO to amplify a DNA fragment of 600-bp downstream of the gene; and primers C-PK-NEO-KO and primer D-PK-NEO-KO, to amplify the *NEO* gene. The fragment generated was cloned in the pGEM-T-easy vector leading to the pGEM-T-KO-NEO-PK. The LmjF30.1250 inactivation cassette was isolated from pGEM-KO-NEO-PK by an *Eco*RI digestion and transfected in *L. major* Friedlin. A second inactivation cassette containing a HYG marker was generated using the A and F primers described above along with primers B-PK-HYG-KO, D-PK-HYG-KO and E-PK-HYG-KO (Table S1). The fragment generated was cloned in the pGEM-T-easy vector leading to the second construct pGEM-T-KO-HYG-PK to inactivate the *PK* gene. The integration of the inactivation cassette at the LmjF30.1250 locus was confirmed by PCR and Southern blots. A rescue vector containing the wild-type gene was generated by PCR amplification using the primers PKF and PKR, cloned in pGEM-T easy vector, sequenced and then subcloned in pSP72αZEOα generating the plasmid pSP72αZEOα-PK. All the primers used are listed in Table S1.

### Genome resequencing and analysis

Genomic DNAs were prepared from mid-log phase cultures of *L. major* Friedlin mutants MF80.3 and MF80.5 and *L. major* Friedlin wild-type parasites and sequenced using Illumina Genome AnalyzerIIx short 76-nucleotide single-end reads. We had a genome coverage over 50-fold for the two independent mutants and the wild-type parasite. This strategy allowed us to identify point mutations and small indels (≤3 bp) when comparing with the known genome sequence of *L. major* Friedlin [34].

### Data analysis

Sequence reads from each clone were aligned to the *L. major* Friedlin reference sequence available (TriTrypDB version 3.3) [35] using the software bwa (bwa aln, version 0.5.9) with default parameters [36]. The maximum number of mismatches was 4, the seed length was 32 and 2 mismatches were allowed within the seed. The detection of single nucleotide polymorphisms (SNPs) was performed using samtools (version 0.1.18), bcftools (distributed with samtools) and vcfutils.pl (distributed with samtools) [37], with a minimum of three reads to call a potential variation prior to further analysis. The sequence data for *L. major* Friedlin wild-type, MF80.3 and MF80.5 are available at the EMBL European Nucleotide Archive (http://www.ebi.ac.uk/ena) (accession number ERP000917; samples ERS056015, ERS056016 and ERS056017 corresponding to *L. major* Friedlin wild-type; MF80.3 and MF80.5 respectively). Several python (version 2.4.3) scripts and bash (version 3.2) scripts were created to further analyse the data. The quality assessment software samstat (v1.08) was used to generate quality reports [38].

All the putative point mutations detected by whole genome sequencing were verified by PCR amplification and conventional DNA sequencing. The primers were designed using Primer3 program [39].

## Results

### Characterization of *L. major* Friedlin MF-resistant mutants

**Figure 1:**
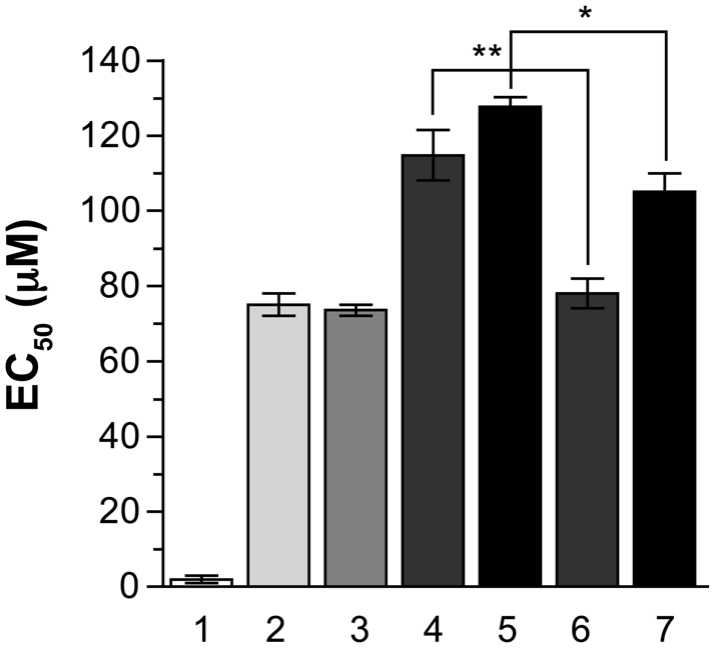
Miltefosine susceptibilities of *Leishmania major* mutants and wild-type parasites. The EC_50_ values were determined by culturing promastigote parasites in the presence of increasing concentrations of MF. The average of three independent experiments is shown. EC_50_ values for MF in *L. major* wild-type parasites (1); MF80.1 (2); MF80.2 (3);MF80.3 (4); MF80.5 (5) MF-resistant mutants. The MF80.3 and MF80.5 mutants were grown in the absence of MF for 30 passages (6 and 7 respectively). * p<0.05; ** p<0.01.

Four independent *L. major* Friedlin MF-resistant mutants (MF80.1, 80.2, 80.3 and 80.5) were generated by a stepwise fashion until they reached a MF EC_50_ of at least 80 µM (Fig. 1). The MF80.3 and MF80.5 lines displayed the highest levels of resistance, with at least 15 fold increase in resistance compared to the wild-type (WT) parent. The phenotype of MF resistance appeared relatively stable, as culturing the parasites in absence of MF for 30 passages led to only a small albeit significant decrease in resistance in both MF80.3 and MF80.5 (Fig. 1, lanes 6, 7) but also in MF80.1 and MF80.2 (data not shown). Because of the small decrease of resistance, we tested whether MF resistance in our mutants was accompanied by gene amplification, a frequent mechanism of drug resistance in *Leishmania* [40], [41]. However, neither alkaline lysis nor clamped homogeneous electrical field electrophoresis revealed the presence of circular or linear amplicons in any of MF resistant mutants (results not shown).

### Whole-genome sequencing of *L. major* Friedlin MF-resistant mutants

The genomes of one clone derived from the wild-type *L. major*, MF80.3 and MF80.5 mutants were resequenced using the single-end reads Illumina platform. We obtained a total of 30,083,681, 28,369,175 and 28,259,044 reads leading to an average genome coverage of 52-fold for the wild-type *L. major* Friedlin strain and MF80.3 and MF80.5 respectively. In all cases, close to 80% of the reads were aligned to the *L. major* Friedlin genome. The unmapped reads (25.1%, 20.2% and 18.5% respectively for *L. major* wild-type, MF80.3 and MF80.5) had low mean base quality according to samstat (version 0.08) [38] with slightly less than half mapping to mitochondrial DNA. Read depth coverage can be used to predict copy number variations being either amplifications or deletions. We investigated copy number of chromosomal regions by read depth coverage for each chromosome of the resistant mutants which were compared to the wild-type sensitive *L. major* Friedlin. This analysis did not reveal changes in read depth between sensitive and resistant isolates that would constitute signatures of copy variation, hence confirming the absence of gene amplification in these mutants but also indicating no obvious gene/*locus* deletion or changes in ploidy (data not show). While no change in copy number was related to resistance, we found that some chromosomes were polyploid in both *L. major* Friedlin wild-type cells and in the two MF-resistant clones. Chromosome 1, 5, 11, 20 and 23 were in three copies while chromosome 31 was at least in six copies in the wild-type cells but also in the mutants (Fig. S1A–C).

While no changes in copy number were observed, the resequencing of the *L. major* Friedlin wild-type strain and of the mutants differed from the published sequence [34]. Indeed, 20 homozygous (Table S2) and 42 heterozygous SNPs (Table S3) were observed. The polymorphic sites identified here in the coding sequences were highlighted by resequencing the wild-type Friedlin strain, the two MF-resistant strains and also other *L. major* Friedlin resistant mutants that we are currently analysing in the lab. A majority of polymorphisms (68%) changed the amino acids of the encoded proteins (Table S2 and S3). Thus, the majority of putative mutations found by this next-generation sequencing approach corresponded to natural polymorphisms present in the parental clones and that were not selected for during the drug pressure.

However, whole genome sequencing of the two miltefosine resistant mutant clones also revealed genuine mutations (Table 1). Indeed, a three nucleotide deletion was seen in in the *MT* gene of MF80.3 (LmjF13.1530) and a transition mutation leading to a mutated G852D protein version was observed in mutant MF80.5 (Table 1). These mutations highlighted by next generation sequencing were confirmed by PCR amplification and standard DNA sequencing and the same validation approach was used for all other mutations that will be discussed afterwards. Interestingly, another mutation was in a common gene for both mutants. It corresponded to the LmjF30.1250 gene that code for a putative pyridoxal kinase (PK). The MF80.3 mutant acquired a mutation in both alleles that led to a T216M amino acid substitution while one wild-type allele and one allelic version leading to a G269D protein were observed in the MF80.5 line (Table 1). Other mutations were specific to either MF80.3 or MF80.5. Three heterozygous mutations in LmjF15.0800 and LmjF.27.1250, both coding for hypothetical proteins, and in LmjF36.5880 coding for a small GTPase were found in MF80.3 (Table 1). The sequences of the MF80.5 clone allowed the detection of a homozygous mutation in LmjF07.0050 coding for an α-adaptin like protein and an heterozygous mutation in the hypothetical protein coding gene LmjF21.0835 (Table 1).

**Table 1:** Gene mutations identified in *L. major* miltefosine-resistant mutants.

A. MF80.3								
Chromosome	Type	Start	End	Reference base in GeneDB	Base in MF80.3	*L. major* gene ID	Position in the gene	Amino acid change
13	Deletion	567388	567391	TGA	-TGA	LmjF13.1530	1640–1642	M547del
15	SNP		368510	C	C or T	LmjF15.0800	1531	H511Y
27	SNP		526263	C	C or G	LmjF27.1250	407	T136R
30	SNP		427336	C	T	LmjF30.1250	647	T216M
36	SNP		2256582	C	C or G	LmjF36.5880	537	F179L

B. MF80.5								
Chromosome	Type	Start	End	Reference base in GeneDB	Base in MF80.5	*L. major* gene ID	Position in the gene	Amino acid change
7	SNP		19641	G	C	LmjF07.0050	2284	D762H
13	SNP		566476	C	T	LmjF13.1530	2555	G852D
21	SNP		305528	C	C or G	LmjF21.0835	868	L290V
30	SNP		427495	G	G or A	LmjF30.1250	806	G269D

Summary of all mutations (single nucleotide mutations (SNPs) and indels) identified in clones derived from *L. major* MF80.3 (A) and MF80.5 (B) miltefosine resistant mutants using the software BWA and Samtools.

Since few genuine point mutations were highlighted by whole genome sequencing, we next amplified and sequenced these specific mutated genes in additional *L. major* and *L. infantum* MF resistant mutants that were available. This targeted approach revealed that only the *MT* and *PK* genes were mutated in the four independent *L. major* resistant mutants (Table 2), and that *MT* was also mutated in the two *L. infantum* mutants investigated (Table 2). The mutations were at different sites in the *L. infantum* or *L. major* mutants (Table 2), but these mutations occurred at conserved sites within the MT protein (Fig. S2). The targeted sequencing of the *PK* gene in the other *L. major* mutants revealed that they were also heterozygous for mutations leading to G269D or T216M amino acid substitutions, respectively (Table 2). However, the *PK* gene was not mutated in the two *L. infantum* MF resistant mutants analysed (Table 2). The LmjF07.0050 α-adaptin gene was the only other mutation that was not strain-specific since the gene was also mutated in the two independent *L. infantum* MF resistant mutants (Table 2 and Fig. S3). The four remaining mutations highlighted by next-generation sequencing were unique to either MF80.3 or MF80.5 (Table 2). Surprisingly, we did not observe point mutations in the Ros3 β-subunit of MT, a known MF-resistance gene [12], in any of the miltefosine resistant mutants analyzed (Table 2).

**Table 2:** Mutations potentially involved in miltefosine resistance in *Leishmania*.

Genes / mutants	MF80.1	MF80.2	MF80.3	MF80.5	Li MF200.3	Li MF200.5
Miltefosine transporter (*LmjF13.1530*)	P782T	W895*	M547del	G852D	W617*	G565R
Ros3 (*LmjF32.0510*)	No	No	No	No	No	No
Pyridoxal kinase (*LmjF30.1250*)	G269G G269D	T216T T216M	T216M	G269G G269D	No	No
α-Adaptin like protein (*LmjF07.0050*)	No	No	No	D762H	C54F	F58L
Hypothetical protein (*LmjF15.0800*)	ND	ND	H511H H511Y	No	ND	ND
Hypothetical protein (*LmjF21.0835*)	No	No	No	L290L L290V	No orthologue in *L. infantum*	No orthologue in *L. infantum*
Hypothetical protein (*LmjF27.1250*)	No	No	T136T T136R	No	No	No
Small GTPase (*LmjF36.5880*)	No	No	F179F F179L	No	No	No

*L. major* and *L. infantum* (Li) genes potentially involved in miltefosine (MF) resistance were sequenced. Heterozygous and homozygous mutations are indicated.

*- Stop codon. No – no mutation. ND – Not done.

**Figure 2:**
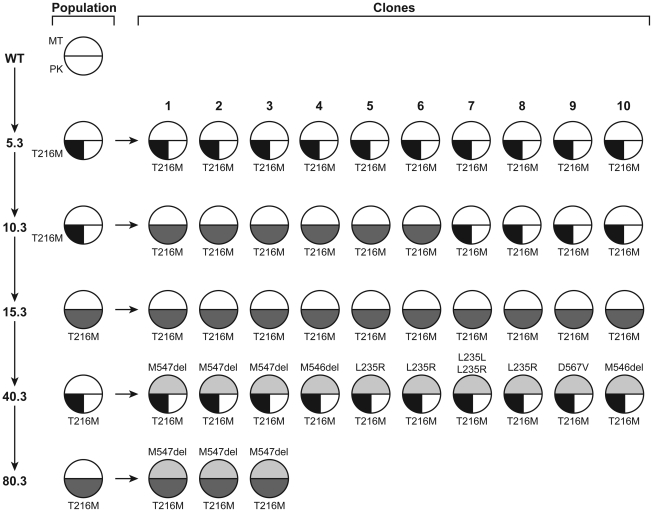
Mutations in the miltefosine transporter (MT) and pyridoxal kinase (PK) in the MF80.3 lineage. The top and bottom half circle represents the *MT* and *PK* genes respectively. White represents wild-type sequences; semi-colored an heterozygous mutation and fully colored an homozygous mutation. The type of mutation is also indicated.

We were intrigued by the diversity of mutations in the *MT* gene between the different mutants (Table 2) suggesting that mutations in MT could be polyclonal. We investigated the MT status in clones (and population) isolated at intermediate passages leading to the generation of MF80.3. Most interestingly, no mutations in MT were found in any of the resistant populations leading to MF80.3 (Fig. 2), suggesting indeed that resistance can be polyclonal, and since no clones are dominant, mutations can be missed when sequencing the population. In none of the 10 clones investigated derived from MF5.3, MF10.3, or MF15.3 could we detect any mutations in MT. However in the 10 clones derived from MF40.3 where the *MT* gene was sequenced, all clones had a mutated MT and 5 different genotypes were observed. Three clones had a three nucleotide deletion leading to the deletion of M547, two had a deletion of M546, one clone had a D567V mutation and 4 clones had a L235R mutation (3 homozygous and one heterozygous) (Fig. 2). The three clones derived from MF80.3 had the deletion of M547 (Fig. 2). The situation was different in the lineage leading to MF80.5. Indeed, the homozygous G852D mutation found in the MT of the sequenced clone (Table 1) was also detected in the MF80.5 population but also in the MF60.5 population and the mutation was heterozygous at the earlier passages of MF20.5 and MF40.5 (Table 3). Similarly to the lineage leading to MF80.3, mutations in the *MT* gene were observed only at the highest drug concentration. In contrast to MT, mutations in the *PK* gene LmjF30.1250 appeared during the first selection of MF selection in both MF80.3 and MF80.5 mutants (Fig. 2 and Table 3). While the mutation remained heterozygous in the MF80.5 lineage (Table 3), the number of mutated alleles in the *PK* gene in the MF80.3 lineage, with the exception of MF40.3 (see below), correlated with resistance levels (Fig. 2).

**Table 3:** Mutations potentially involved in miltefosine resistance in the MF80.5 lineage.

Mutants/genes	Miltefosine transporter (*LmjF13.1530*)	Pyridoxal kinase (*LmjF30.1250*)	α-adaptin like protein (*LmjF07.0050*)
MF5.5	No mutation	G269D/G269G	No mutation
MF10.5	No mutation	G269D/G269G	No mutation
MF20.5	G852D/G852G	G269D/G269G	No mutation
MF40.5	G852D/G852G	G269D/G269G	No mutation
MF60.5	G852D	G269D/G269G	No mutation
MF80.5	G852D	G269D/G269G	D762H

Heterozygous and homozygous mutations are indicated.

### Functional analysis of genes involved in MF resistance

**Figure 3:**
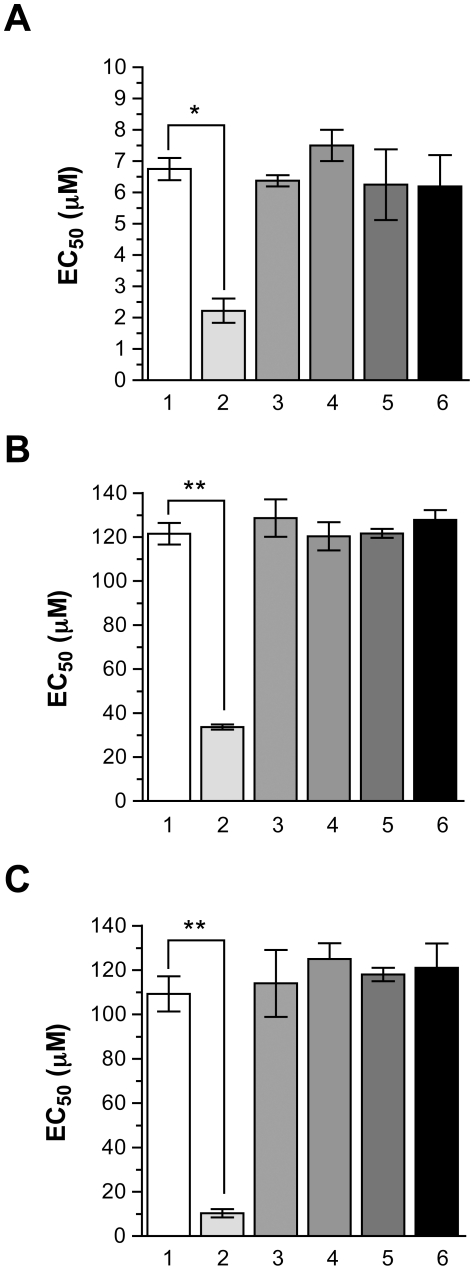
Miltefosine susceptibilities of *Leishmania major* wild-type and MF-resistant transfectants. The EC_50_ values were determined by culturing promastigote parasites in the presence of increasing concentrations of MF. The average of three independent experiments is shown. LmjF WT (A), MF80.3 (B) and MF80.5 (C) parasites were transfected with pSP72αHYGα (1); pSP72αHYGα-MT (2); pSP72αHYGα-MT* (G852D) (3); pSP72αHYGα-MT* (M547del) (4); pSP72αNEOα-PK (5); and pSP72αHYGα-adaptin like protein (6). * p<0.01; ** p<0.001.

The role in resistance of the *MT* mutations highlighted in one clone of MF80.3 and MF80.5 was further studied by gene transfection experiments. Episomal transfection of the wild-type copy of *MT* sensitized wild-type cells to MF by 3-fold whereas transfections of the G852D or M547- versions cloned in the same vector did not change the phenotype (Fig. 3A, bars 2–4). Transfection of the wild-type gene copy of MT in MF80.3 and MF80.5 sensitized the mutants to MF although not to wild-type levels (Fig. 3B and C, bar 2). Significantly, transfection of the mutated versions of *MT* neither sensitized the mutant cells nor the wild-type cells, showing that these mutations alter the function of MT (Fig. 3, bars 3, 4).

The role of the PK and α-adaptin-like proteins that were mutated in more than one mutant (Table 1), in MF resistance was also assessed by gene transfection experiments. In contrast to the *MT* gene, however, the overexpression of WT versions of the *PK* or α-adaptin-like genes failed to alter the MF resistance levels of *L. major* WT (Fig. 3A, bars 5 and 6), MF80.3 (Fig. 3B, bars 5 and 6) and MF80.5 mutants (Fig. 3C, bars 5 and 6). Transfection of mutated versions of the *PK* and α-adaptin-like genes also failed to alter the MF susceptibility status of the wild-type *L. major* Friedlin strain (data not shown). The *PK* mutations were acquired during the first step of selection in both lines, prior to the selection of mutations in the MT (Fig. 2 and Table 3). To further investigate the role of PK in MF resistance, we investigated clones at different level of selection giving rise to MF80.3. The genotype of PK varied with clones being either heterozygous (WT/T216M) or T216M homozygous mutants (Fig. 2). The level of resistance to MF and the number of mutated PK alleles was not straightforward but a wild-type PK allele has never been found in MF resistant cells (Fig. 2, and Table 3). Only heterozygous mutations were found in clones derived from MF5.3 but in MF10.3 we found a mixture of clones with either homozygous or heterozygous mutations in PK, while all clones derived from MF15.3 had homozygous T216M mutation in PK (Fig. 2). In the MF40.3 clone, we observed cells that were surprisingly heterozygous for PK but these cells have a mutation in MT, a mutation not present in the MF15.3 derived clones (Fig. 2) and possibly the establishment of mutations in MT is facilitated in cells still having some wild-type PK activity. When the MF40.3 population was selected for higher resistance to yield MF80.3, all clones exhibited the same PK homozygous mutation (Fig. 2).

**Figure 4:**
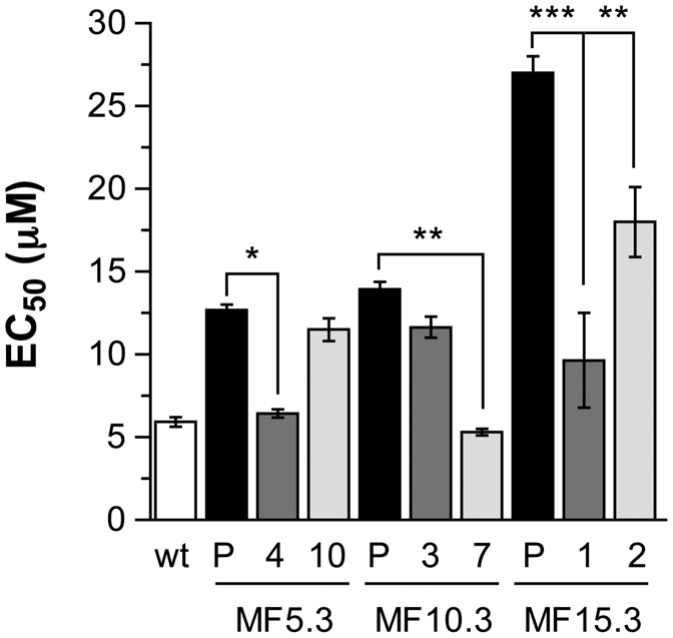
Miltefosine EC_50_ values of *L. major* parasites. Drug susceptibility of wild-type cells (white bars) and MF-resistant mutant population (P) or clones (gray shaded bars) were measured. The clones were derived from MF5.3, MF10.3 and MF15.3 population and clone numbers are relate to Fig. 2. The average of three independent measures is shown. * p<0.05; ** p<0.01; *** p<0.001.

We also investigated the susceptibility levels of selected clones for the earlier passages. While, as expected, the population of MF5.3, MF10.3 and MF15.3 became more and more resistant to MF (Fig. 4) we found variations in the EC_50_ between the individual clones derived from the population. Indeed, some clones were as resistant as the population and others had susceptibilities close to wild-type levels (Fig. 4). For example, clones 4 and 10 derived from MF5.3 had the same genotype (for MT and PK) but clone 4 had an EC_50_ close to wild-type while clone 10 was resistant to MF (Fig. 4). Similarly, the cloning of MF10.3 led to parasite with either homozygous (clone 3) or heterozygous (clone 7) mutations in PK and clone 3 was found to be more resistant (Fig. 4). However, clones 1 and 2 derived from MF15.3 had the same genotype (for MT and PK) but their resistance to MF varied (Fig. 4).

**Figure 5:**
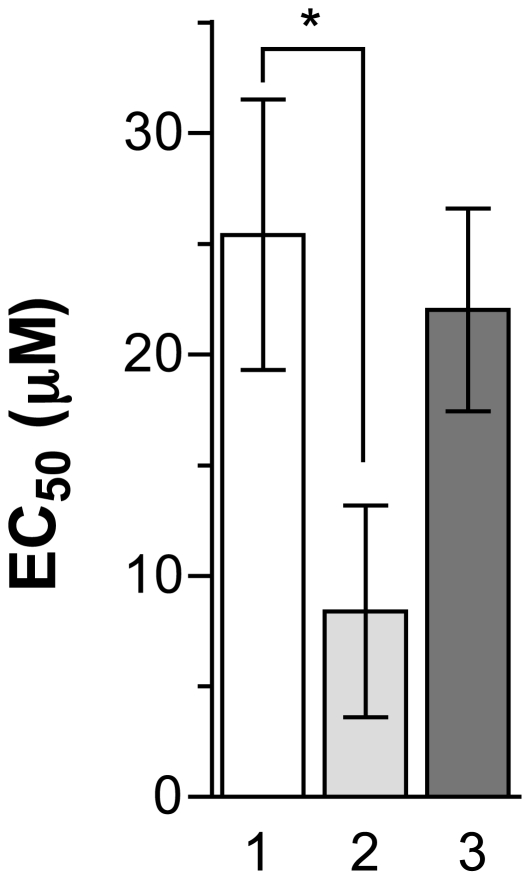
Miltefosine susceptibilities of *L. major* parasites transfected with the *PK* gene. The EC_50_ values were determined by culturing promastigotes parasites in the presence of increasing concentrations of MF. The average of three independent experiments is shown. *L. major* MF15.3 transfected with the empty vector pSP72αNEOα (1); pSP72αNEOα-PK (2); or pSP72αNEOα-PK* (T216M) (3). * p<0.05.

**Figure 6:**
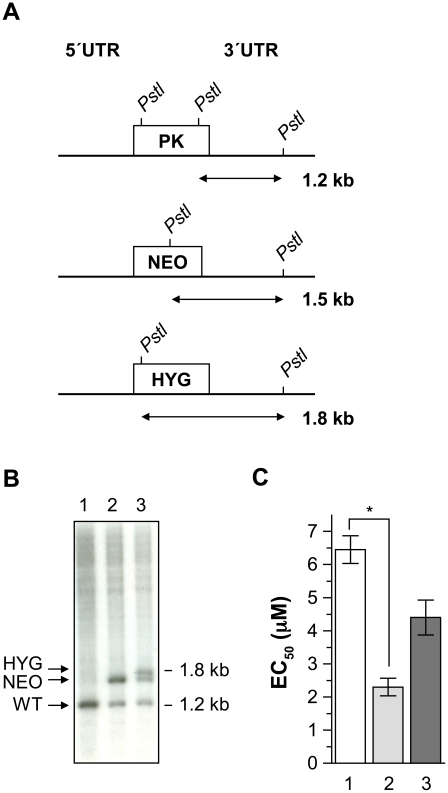
Inactivation attempts of the *L. major PK* gene. (A) A schematic drawing of the PK locus with *Pst*I sites. (B) Southern blot analysis of *L. major* genomic DNA digested with *Pst*I and hybridized with a 3′ UTR probe of *PK* gene (a 600 bp fragment just upstream the stop codon of *PK* gene). Lane 1, *L. major* wild-type; lane 2, *L. major* with one PK allele disrupted with the NEO marker; lane 3, *L. major* with two alleles disrupted (HYG and NEO). Molecular weight is indicated on the right and the alleles are indicated on the left. (C) Miltefosine susceptibilities of *Leishmania major* wild-type (1), SKO-PK (2) and SKO-PK complemented with a wild-type *PK* gene (3) parasites. The EC_50_ values were determined by culturing promastigotes parasites in the presence of increasing concentrations of MF. The average of three independent experiments is shown. * p<0.01.

While no susceptibility phenotype was observed in transfecting PK in MF80.3 (Fig. 3B), the MF15.3 mutant is homozygous for the T216M PK substitution and still has a wild-type version of MT (Fig. 2). The transfection of the wild-type PK-containing plasmid in MF15.3 resulted in the reversion of MF resistance (Fig. 5). In contrast, the transfection of theT216M version had no effect on MF susceptibility (Fig. 5). We next investigated whether deletion of *PK* was possible and tested how this could modulate MF susceptibility. Two replacement cassettes containing the neomycin phosphotransferase (*NEO*) or hygromycin phosphotransferase (*HYG*) genes were generated for replacing PK (Fig. 6). Since *Leishmania* is diploid, the sequential integration of the *NEO* or *HYG* inactivation cassette should lead to a 1.5 and 1.8 kb DNA fragment, respectively, in addition to the remaining WT *PK* fragment of 1.2 kb when the DNA of transformants is digested with *Pst*I and hybridized to a 3′UTR *PK* probe corresponding to a 600 bp fragment upstream the *PK* gene stop codon (Fig. 6A). The generation of NEO single disruptant led to the expected genotype (Fig. 6B, lane 2). Introduction of the *HYG* construct into a *PK/NEO* parasite indicated that while both *NEO* and *HYG* alleles integrated properly, there was always a remaining wild-type allele (Fig. 6B, lane 3), a phenomenon often observed when attempting to inactivate essential genes [42]–[45]. Parasites with a single allele of *PK* inactivated with NEO were more sensitive to MF than the control-transfected parasites (Fig. 6C) and this phenotype could be partially rescued by the addition of an episomal construct encoding a functional copy of PK (Fig. 6C).

## Discussion

We sequenced two *L. major* MF-resistant mutants in order to identify mutations putatively associated with MF resistance. Our strategy was based on sequencing two independent resistant strains and to assess the role in resistance of recurrent mutations. *Leishmania* often amplifies regions of its genome in response to drug selection [40], [41], but this does not appear to be the case for the MF resistant strains as determined by read depth sequence analysis (Fig. S1A–C) and also further confirmed by comparative genomic hybridization experiments (unpublished observations). The absence of amplicons in our MF-resistant *L. major* is consistent with the previous characterization of MF resistant strains of *L. donovani* [25]. However, the analysis revealed the presence of point mutations in protein coding genes for both *L. major* MF-resistant mutants (Table 1). Three mutations were found in more than one mutant: the miltefosine transporter MT LmjF13.1530, the pyridoxal kinase PK LmjF30.1250, and the α-adaptin like protein LmjF07.0050 (Table 2).

The MT protein is a P-type ATPase that mediates the inward translocation of MF and glycerophospholipid analogues in *Leishmania* [9], [12]. The acquisition of inactivating mutations in the *MT* gene was shown to be a major determinant of resistance to MF in *L. donovani* and it appears that several distinct mutations in *MT* can confer resistance in both promastigote and amastigotes parasites [9], [20]. The mutant Li MF200.5 with the *MT* gene mutated, was indeed shown to be MF resistant using an intramacrophagic essay (results not shown). Although several distinct *MT* point mutations have also been identified in our panel of *L. major* MF-resistant mutants (Table 2), they all differed from those previously reported in *L. donovani*. These mutations were located at conserved residues within the P-type ATPase protein (Fig. S2). The number of mutated alleles in MT correlated with the level of resistance to MF with the most resistant strains being homozygous mutants. Mutations in MT only occur at later step of drug selection (Fig. 2, Table 3).

Prior to MT mutations, the only other recurring mutation in low-level resistant cells was the acquisition of the T216M or G269D amino acid substitutions in the *PK* gene. Similarly as for MT, the level of MF resistance correlated with the number of mutated *PK* alleles in the MF 80.3 lineage (Fig. 2). As explained in the result section, it is possible that mutations in *MT* happens in a minority population of cells with heterozygous *PK* alleles and this could explain how cells appear to be mostly homozygous for a mutant allele in *PK* (15.3), and then becomes heterozygous (40.3).. Pyridoxal-5′-phosphate (PLP) is the active form of vitamin B6 and is an essential cofactor of several enzymes, predominantly involved in the transformation of amino acids [46], [47]. *Leishmania* cannot synthesize PLP *de novo* and rely on the scavenging of vitamin B6 precursors like pyridoxal, pyridoxine and pyridoxamine [48]. PK was shown to catalyze the ATP-dependent phosphorylation of these precursors and to play an essential role in the formation of the biologically active PLP [46]. The role of PK in MF susceptibility warrants further studies and may be due to the many roles of PLP in cell physiology. Point mutations in *PK* are found in the four *L. major* MF-resistant mutants (Table 2) and transfection of a wild-type gene in MF15.3 restored susceptibility (Fig. 5). Mutations in PK were not observed in MF resistant *L. infantum* (Table 2), possibly suggesting additional species-specific differences. For example, the expression of the MF translocation machinery was found to vary between species and this altered their MF susceptibilities [15]. Mutations in PK were in conserved regions between *Leishmania* and other species but not in active sites (Fig. S4), suggesting that the mutated PK possibly retains activity. These mutations may modulate activities necessary to mitigate the effect of MF. Reintroduction of wild-type alleles eliminates this selective advantage. Our inability to generate a PK null mutant may suggest that this gene is essential and cells lacking one allele were shown to be more susceptible showing that PK is not likely to be a target of MF and that other activities of PK or some enzymatic reactions requiring PLP are essential to deal with MF stresses. MF was shown to induce cellular events similar to those resulting from alterations in the metabolism of PLP. Indeed, MF was shown to induce altered content of phosphatidylcholine (PC) and phosphatidylethanolamine (PE) in MF-sensitive but not MF-resistant *L. donovani* parasites [19], [49]. Interestingly, vitamin B6 deficiency in rat liver cells was shown to lead to a drastic reduction in the methylation of PE to PC [50]. MF was also shown to induce the accumulation of reactive oxygen species (ROS) in a dose-dependent manner in *Leishmania* and this could be reverted by reduced thiols [27]. The major reduced thiol in *Leishmania* is the glutathione-spermidine conjugate trypanothione. The rate limiting step of spermidine biosynthesis is catalyzed by the PLP-dependent enzyme ornithine decarboxylase [51]. Moreover, vitamin B6 has recently been associated with resistance to oxidative stresses and to be itself an efficient scavenger of ROS [52]. Mutations in PK may thus help in sustaining MF pressure by maintaining the PLP levels, the homeostasis of phospholipids, or the redox state of the parasite. Obviously, further experiments will be required to confirm these hypotheses.

Other mutations were identified in the mutants MF80.3 and MF80.5 but these were specific to a mutant with the exception of the α-adaptin protein where mutations were also observed in two *L. infantum* mutants (Table 2). However, these mutations occurred only in the latest step of selection and transfection of the α-adaptin gene did not change MF susceptibility (Fig. 3). While most of the other mutated genes code for hypothetical proteins and are found in single mutants they may be worth further investigating in additional studies.

This study highlighted a number of concepts of general interest in the genesis of drug resistance with also possible practical implications. Resistance appears to be highly heterogeneous at the level of the population with individual clones within the population differing both in terms of genotype (Fig. 2) but also phenotype (Fig. 4). This may be similar to the recent description of bacterial charity where in a resistant bacterial population there are clones with variable susceptibility, various clones helping others depending on the selective pressure [53]. This heterogeneity both in genotype and phenotype in resistant populations may explain the difficulties when studying field isolates. Analysis of drug resistance in several clones may be more revealing than population analysis. Indeed, the sequencing of the MF40.3 (or MF80.3) population failed to highlight mutations in MT but the sequencing of several clones showed mutations associated with resistance (Fig. 2). This study also showed the sequential order of appearance of mutations, with mutations during the selection of resistance in PK arising always before mutations in MT and heterozygous mutation being more frequent in the lower resistant cells and homozygous mutations prevailing in more resistant isolates.

Whole genome sequencing is becoming a useful technique to study resistance in bacteria [54]–[58] and presently applied to the larger parasite genomes [59], [60]. This study pinpointed known and new resistance markers and also allowed the demonstration of the polyclonal nature of a resistant population with individual cells with varying susceptibility and genotypes. In further studies we hope to study more deeply the clonal variety within a resistant population and how these contribute to either resistance or fitness, as recently highlighted for a population of resistant bacteria [53].

## Supporting Information

Figure S1 **Copy number sequencing analysis for all** ***Leishmania*** **chromosomes for** ***L. major*** **Friedlin wild-type parasites (A) and the mutants MF80.3 (B) and MF80.5 (C).** The 36 chromosomes have been mapped as circles representing the normalized read counts for each chromosome (blue line). For the MF80.3 and MF80.5 mutants, the log2 ratios of normalized read counts compared to the *L. major* Friedlin wild-type reference are shown as black lines. Log2 ratios of at least 1 are shown in green and log2 ratios of at least −1 are shown in red. Methods - Sequence reads were aligned with BWA (Burrow-Wheeler Alignment) [36] and alignments were outputted in the SAM (Sequence Alignment/Map) format [37]. Read alignment positions were grouped in 4 kb windows [61] and the number of reads in each window was normalized by multiplying it with the ratio of the total number of reads for the *L. major* Friedlin wild-type compared to the total number of reads obtained for the corresponding resistant mutant. This allowed us to remove the bias associated to the different number of reads per sequencing run. For each sample, a ratio was computed for each window in respect to the corresponding window in the reference sample (to remove some biases such as GC bias). The logarithm in base 2 was then computed for each ratio to reduce the span of observed values and to obtain a continuous spectrum [62]. Data was plotted in a circular fashion using Circos, a versatile information-aesthetic framework for data visualisation [63]. Log-2 ratios of at least 1 are shown in green whereas of at least −1 are shown in red.
(TIF)

Figure S2 **Protein sequence alignment of** ***L. major*** **MT (LmjF13.1530) with its respective orthologs of** ***L. infantum*** **(LinJ13_V3.1590) and** ***L. braziliensis*** **(LbrM13_V2.1380).** Alignment was performed using the ClustalW algorithm implemented in the Lasergene software (DNASTAR, Inc.). Identical residues are shaded in black. The amino acids mutated in the MT of *L. major* MF mutants are marked by the lower arrowheads.
(TIF)

Figure S3 **Protein sequence alignment of** ***L. major*** **α-adaptin like protein (LmjF07.0050) with its respective orthologs of** ***L. infantum*** **(LinJ07_V3.0060) and** ***L. braziliensis*** **(LbrM7_V2.0050).** Alignment was performed using the ClustalW algorithm implemented in the Lasergene software (DNASTAR, Inc.). Identical residues are shaded in black. The amino acids mutated in the α-adaptin like protein of *L. major* and *L. infantum* MF mutants are marked by the lower arrowheads.
(TIF)

Figure S4 **Protein sequence alignment of** ***L. major*** **PK (LmjF31.1250) with its respective orthologs of** ***L. infantum*** **(LinJ13_V3.1590),** ***L. braziliensis*** **(LbrM13_V2.1380),** ***T. brucei*** **(Tb06.5F5.240),** ***E. coli*** **(NP_416153) and** ***Homo sapiens*** **(NP_003672).** Alignment was performed using the ClustalW algorithm implemented in the Lasergene software (DNASTAR, Inc.). Identical residues are shaded in black. The amino acids mutated in the PK of *L. major* MF mutants are marked by the lower arrowheads.
(TIF)

Table S1 **Primers used in this study for amplifying the genes** ***MT*** **(LmjF13.1530),** ***PK*** **(LmjF30.1250) and the** ***α-adaptin like protein*** **(LmjF07.0050) of** ***L. major*** **.** Restriction sites are underlined. Primers are also listed to sequence the genes *MT*, the α-adaptin like protein and *Ros3* (LmjF32.0510) of *L. major* and to generate PK-KO constructs.
(DOC)

Table S2 **Homozygous SNPs found after resequencing the genome of** ***L. major*** **Friedlin.** All the SNPs were identified in *L. major* Friedlin wild-type and in the mutants MF80.3 and MF80.5. The symbol * corresponds to a potential stop codon.
(DOC)

Table S3 **Heterozygous allelic polymorphisms in** ***L. major*** **Friedlin.** All the same SNPs were identified in *L. major* Friedlin wild-type and in the mutants MF80.3 and MF80.5.
(DOC)
