# Supplementary figures and images for: Multiple Mutations in Heterogeneous Miltefosine-Resistant Leishmania major Population as Determined by Whole Genome Sequencing
Source: PLoS Negl Trop Dis. 2012 Feb 14;6(2):e1512. doi: 10.1371/journal.pntd.0001512 (PMC3279362; doi:10.1371/journal.pntd.0001512)

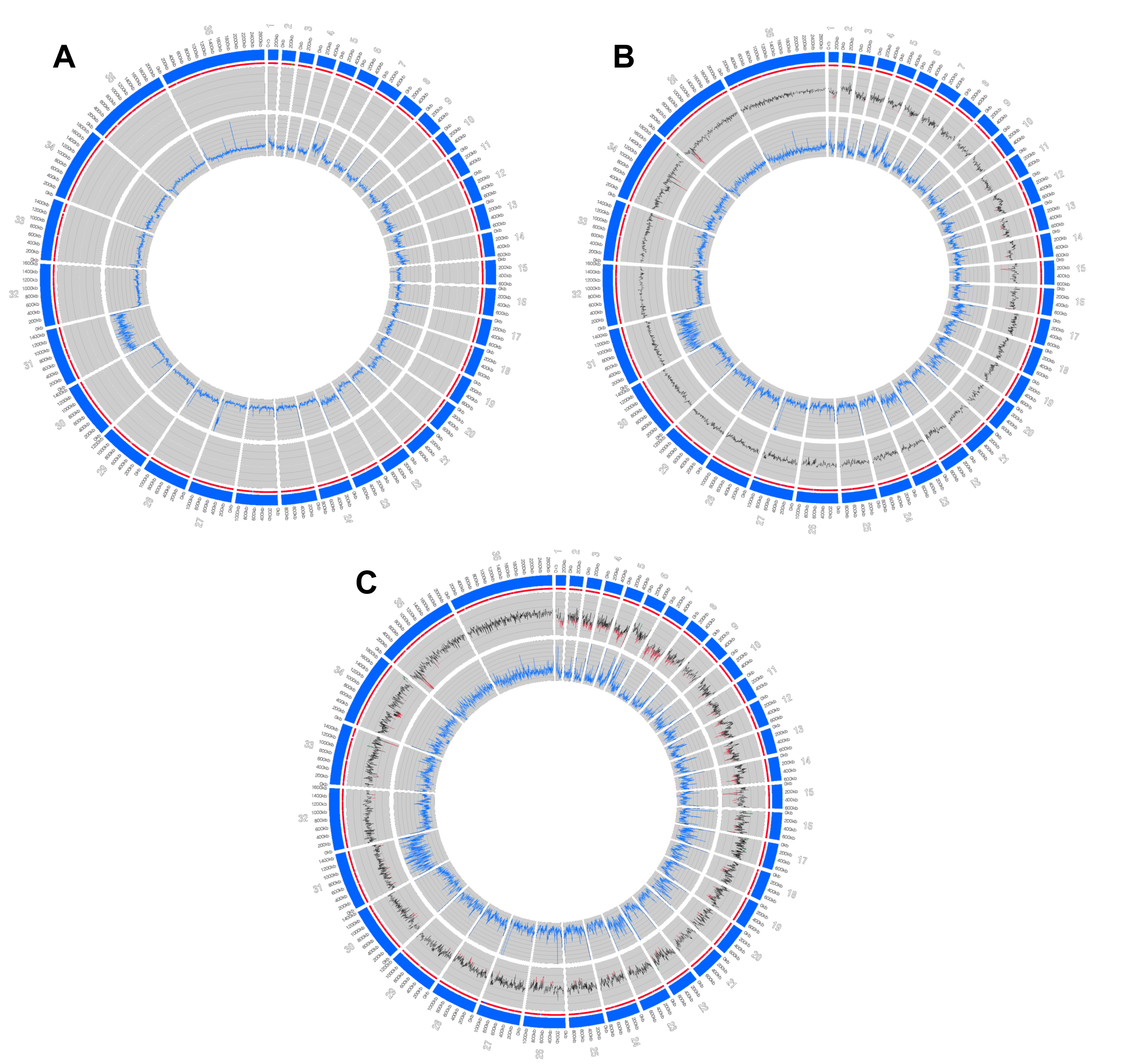

Supplement: Figure S1 — Copy number sequencing analysis for all Leishmania chromosomes for L. major Friedlin wild-type parasites (A) and the mutants MF80.3 (B) and MF80.5 (C). The 36 chromosomes have been mapped as circles representing the normalized read counts for each chromosome (blue line). For the MF80.3 and MF80.5 mutants, the log2 ratios of normalized read counts compared to the L. major Friedlin wild-type reference are shown as black lines. Log2 ratios of at least 1 are shown in green and log2 ratios of at least −1 are shown in red. Methods - Sequence reads were aligned with BWA (Burrow-Wheeler Alignment) [36] and alignments were outputted in the SAM (Sequence Alignment/Map) format [37]. Read alignment positions were grouped in 4 kb windows [61] and the number of reads in each window was normalized by multiplying it with the ratio of the total number of reads for the L. major Friedlin wild-type compared to the total number of reads obtained for the corresponding resistant mutant. This allowed us to remove the bias associated to the different number of reads per sequencing run. For each sample, a ratio was computed for each window in respect to the corresponding window in the reference sample (to remove some biases such as GC bias). The logarithm in base 2 was then computed for each ratio to reduce the span of observed values and to obtain a continuous spectrum [62]. Data was plotted in a circular fashion using Circos, a versatile information-aesthetic framework for data visualisation [63]. Log-2 ratios of at least 1 are shown in green whereas of at least −1 are shown in red. (TIF) [file pntd.0001512.s001.tif]

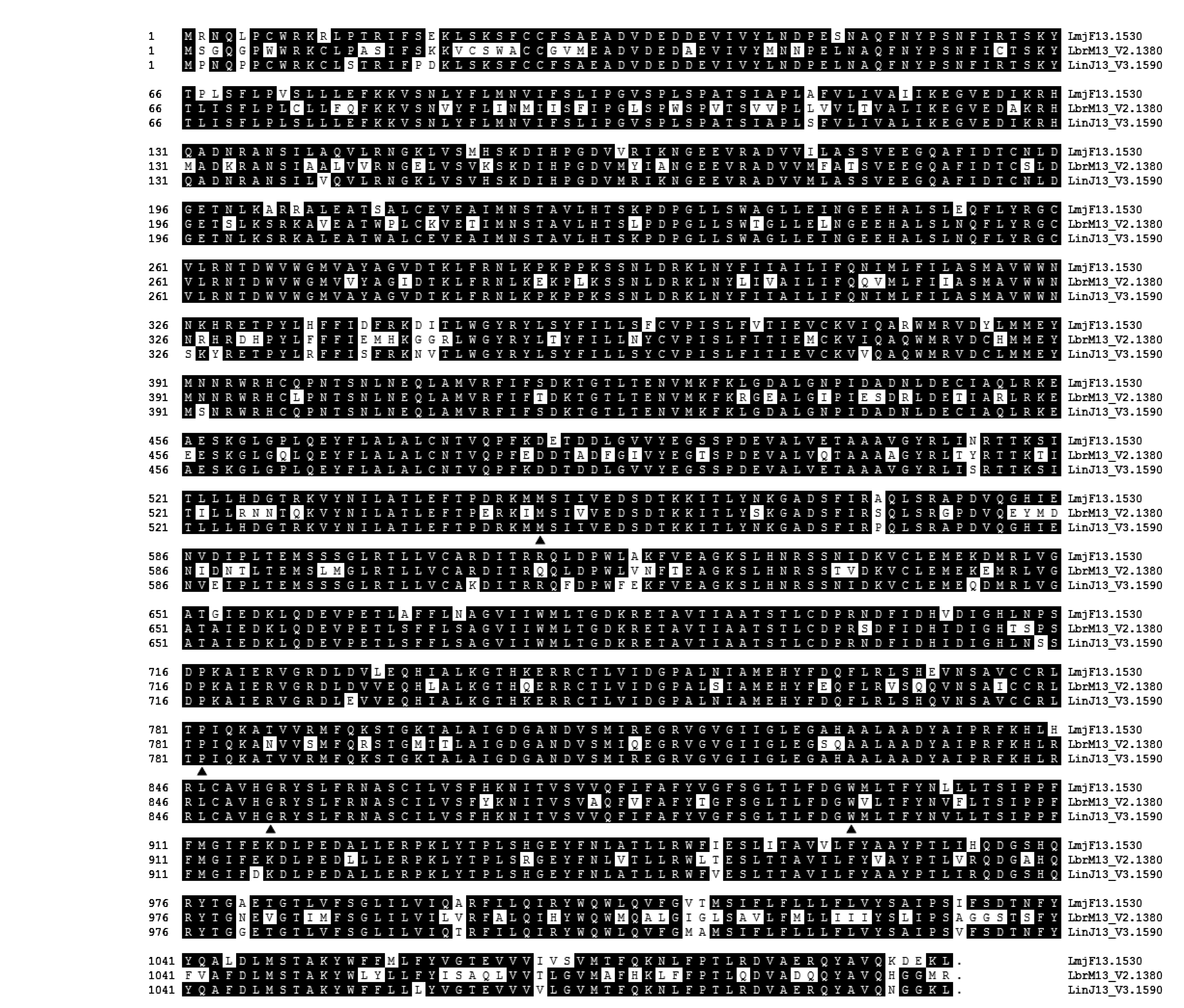

Supplement: Figure S2 — Protein sequence alignment of L. major MT (LmjF13.1530) with its respective orthologs of L. infantum (LinJ13_V3.1590) and L. braziliensis (LbrM13_V2.1380). Alignment was performed using the ClustalW algorithm implemented in the Lasergene software (DNASTAR, Inc.). Identical residues are shaded in black. The amino acids mutated in the MT of L. major MF mutants are marked by the lower arrowheads. (TIF) [file pntd.0001512.s002.tif]

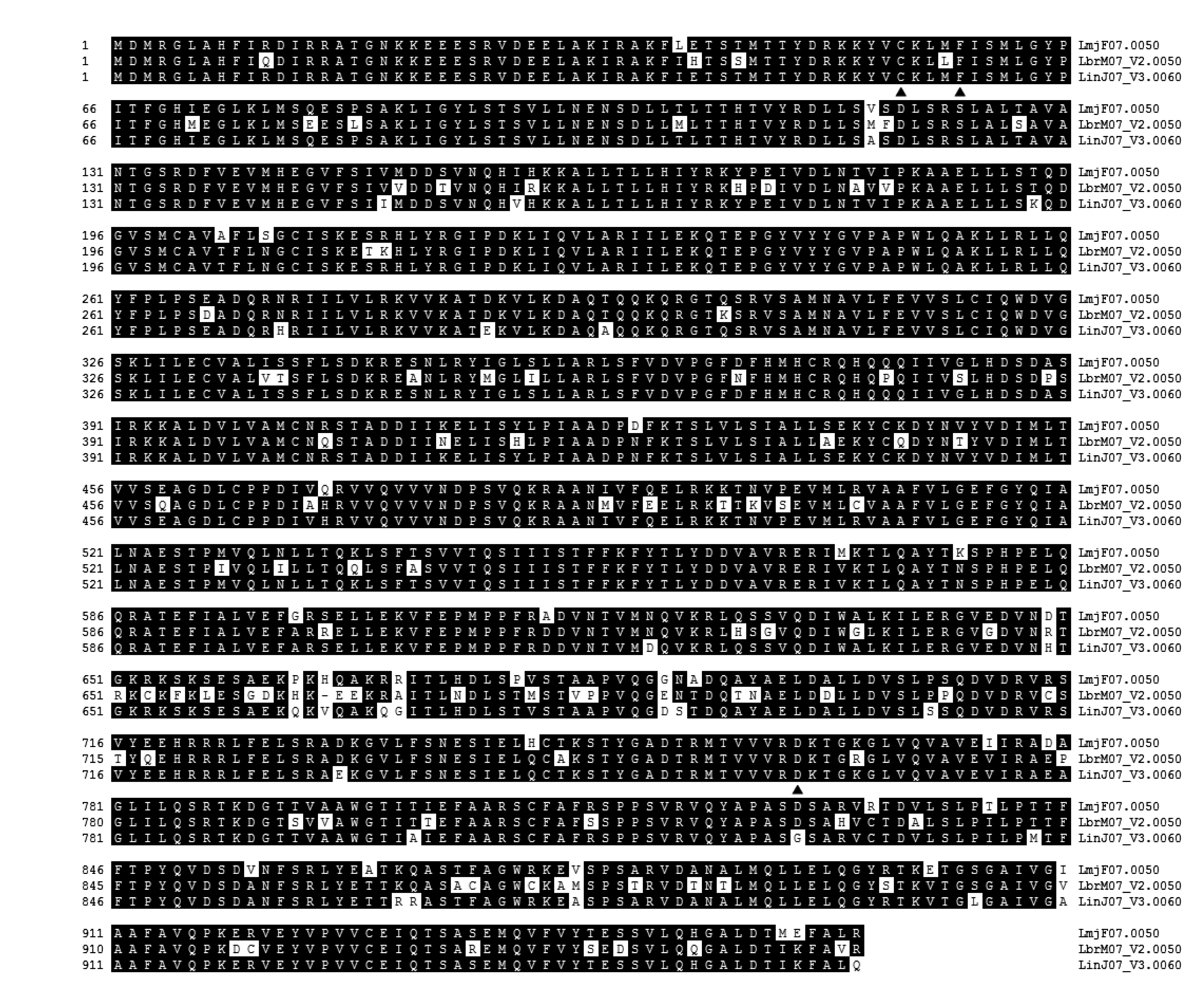

Supplement: Figure S3 — Protein sequence alignment of L. major α-adaptin like protein (LmjF07.0050) with its respective orthologs of L. infantum (LinJ07_V3.0060) and L. braziliensis (LbrM7_V2.0050). Alignment was performed using the ClustalW algorithm implemented in the Lasergene software (DNASTAR, Inc.). Identical residues are shaded in black. The amino acids mutated in the α-adaptin like protein of L. major and L. infantum MF mutants are marked by the lower arrowheads. (TIF) [file pntd.0001512.s003.tif]

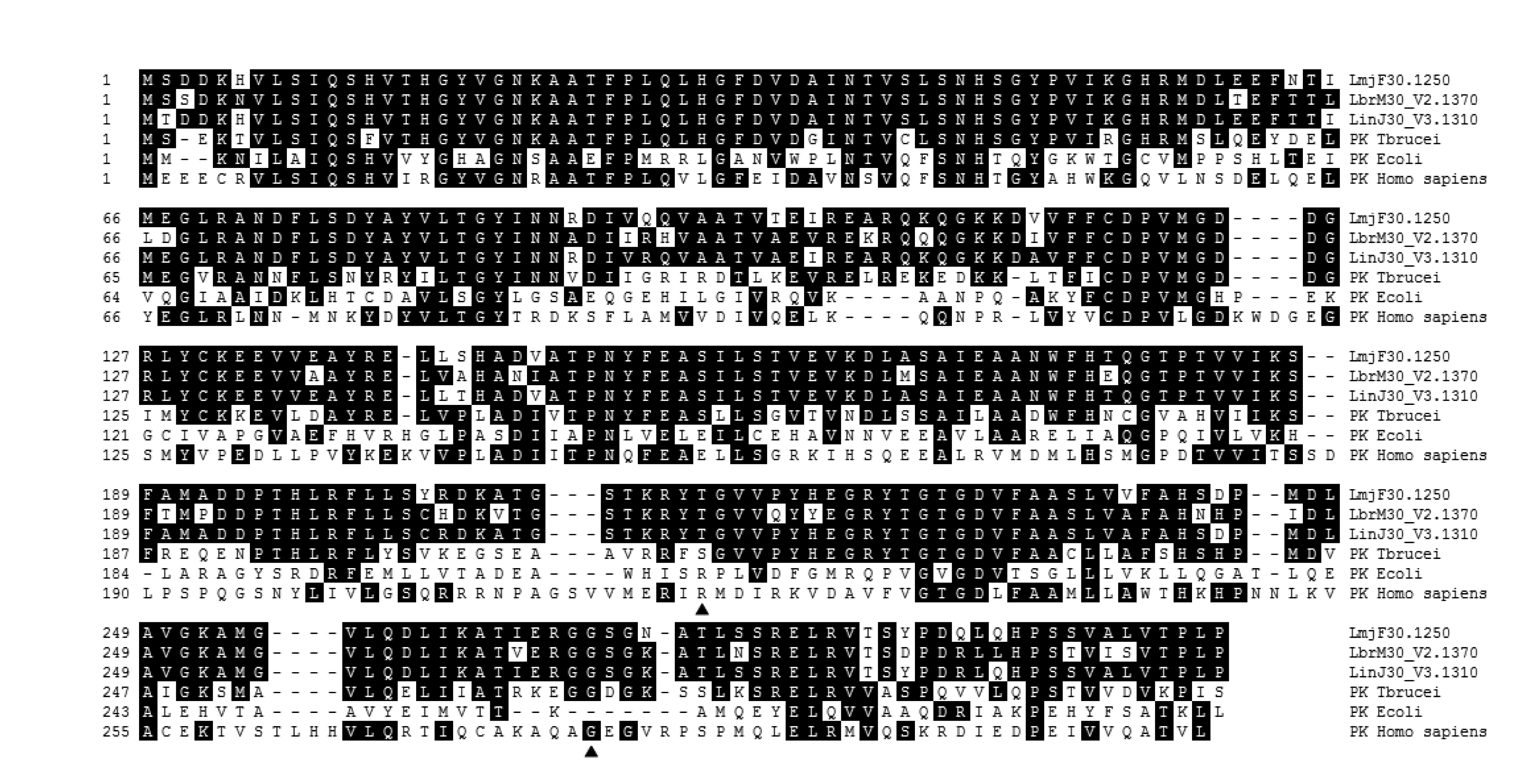

Supplement: Figure S4 — Protein sequence alignment of L. major PK (LmjF31.1250) with its respective orthologs of L. infantum (LinJ13_V3.1590), L. braziliensis (LbrM13_V2.1380), T. brucei (Tb06.5F5.240), E. coli (NP_416153) and Homo sapiens (NP_003672). Alignment was performed using the ClustalW algorithm implemented in the Lasergene software (DNASTAR, Inc.). Identical residues are shaded in black. The amino acids mutated in the PK of L. major MF mutants are marked by the lower arrowheads. (TIF) [file pntd.0001512.s004.tif]
