# Supplementary material for: Multiple Mutations in Heterogeneous Miltefosine-Resistant Leishmania major Population as Determined by Whole Genome Sequencing
Source: PLoS Negl Trop Dis. 2012 Feb 14;6(2):e1512. doi: 10.1371/journal.pntd.0001512 (PMC3279362; doi:10.1371/journal.pntd.0001512)
Supplement: Table S1 — Primers used in this study for amplifying the genes MT (LmjF13.1530), PK (LmjF30.1250) and the α-adaptin like protein (LmjF07.0050) of L. major . Restriction sites are underlined. Primers are also listed to sequence the genes MT, the α-adaptin like protein and Ros3 (LmjF32.0510) of L. major and to generate PK-KO constructs. (DOC) [file pntd.0001512.s005.doc]

### Table S1. Primers used in this study for amplifying the genes *MT* (LmjF13.1530), *PK* (LmjF30.1250) and the *α-adaptin like protein* (LmjF07.0050) of *L. major*. Restriction sites are underlined. Primers are also listed to sequence the genes *MT*, the α-adaptin like protein and *Ros3* (LmjF32.0510) of *L. major* and to generate PK-KO constructs.

| MTF (LmjF13.1530) | 5' GCG TCT AGA GCC CGT CTA TGC GAG TGT ATG |
| --- | --- |
| MTR (LmjF13.1530) | 5' GCG AAG CTT GCC ATG TTC GAT GTG GAT TAG G |
| PKF (LmjF30.1250) | 5' GCG AAG CTT TCC AAC TCT GCG ACT TCC TTG |
| PKR (LmjF30.1250) | 5' GCG TCT AGA AAT CCC TCC CCT GTA CGA CTG |
| α-adaptin protein F (LmjF07.0050) internal | 5' GCG TCT AGA ACC ACC GCT GAC TCT CTG GC |
| α-adaptin protein R (LmjF07.0050) internal | 5' GCG AAG CTT GCA ACG GTT GGA CTC GCA CG |
| α-adaptin protein F2 (LmjF07.0050) internal | 5’ ACTGGCCAAGATACGCGCCA |
| α-adaptin protein F3 (LmjF07.0050) internal | 5’ ATGCACTGCCGTCAGCACCA |
| α-adaptin protein F4 (LmjF07.0050) internal | 5’ AACGTACGGGGCGGACACTC |
| α-adaptin protein R2 (LmjF07.0050) internal | 5’ GCTGCTTCTGCTGCGTCTGC |
| α-adaptin protein R3 (LmjF07.0050) internal | 5’ TTCCGCTTGCCCGTGTCGTT |
| A-PK-KO | 5' TCG GTA CGC TGA GAA CCC GC |
| B-PK-NEO-KO | 5' ACC TGC GTG CAA TCC ATC TTG TTC AAT CAT GCT GCG ACG AAG AGG AGC GA |
| E-PK-NEO-KO | 5' TCG CCT TCT TGA CGA GTT CTT CTG ACA TGC GCC TGC ATC GCT CTG |
| F-PK-KO | 5' GTG CGA GCG CGA GAA AGC AG |
| C-PK-NEO-KO | 5' ATG GGA TCG GCC ATT GAA CA |
| D-PK-NEO-KO | 5' TCA GAA GAA CTC GTC AAG AA |
| B-PK-HYG-KO | 5' GAC GTC GCG GTG AGT TCA GGC TTT TTC ATG CTG CGA CGA AGA GGA GCG A |
| D-PK-HYG-KO | 5' CAG AGC GAT GCA GGC GCA TGC TAT TCC TTT GCC CTC GGA C |
| E-PK-HYG-KO | 5' AGC ACT CGT CCG AGG GCA AAG GAA TAG CAT GCG CCT GCA TCG CTC TG |
| MTF2 | 5’ CAA GCT GAA CTA CTT TAT CA |
| MTR2 | 5’ TGA ACT CCA GTG TGG CGA GG |
| MTF3 | 5’ GGG GCT CCG CAC GCT GCT TG |
| MTR3 | 5’ TCT GGA ACA TGC GAA CGA CG |
| MTF4 | 5’ ATG GCC GCT ACT CGC TCT TC |
| MTR4 | 5’ CAG GTT CGT CTC GCC GTC CA |
| MTF5 | 5' TGC ACA GCA AGG ACA TCC ACC |
| MTR5 | 5' AGC GGT TGT TCA TGT ACT CC |
| MTF6 | 5' TCG ACG CCG ACA ATC TGG ACG |
| MTR6 | 5' CCG ACG AGC CGC ATG TCT TTC |
| MTF7 | 5' CAC GCC GAT CCA GAA GGC AAC |
| MTR7 | 5' ATA CTT GTT AGA AGG AGA TTG |
| Ros3F (UTR) | 5' CGC TCT AGA GTT TTC AGC GGC ATT TTC AC |
| Ros3R (UTR) | 5' CGC AAG CTT TCA GTG CCT AGC AAG AAA AGG |
| Ros3F internal | 5’ TTG CAC TAC TCT CAA CCT CG |
| Ros3R internal | 5’ GTA AAA CTG CTC TAT TGA TG |
