# Supplementary material for: Multiple Mutations in Heterogeneous Miltefosine-Resistant Leishmania major Population as Determined by Whole Genome Sequencing
Source: PLoS Negl Trop Dis. 2012 Feb 14;6(2):e1512. doi: 10.1371/journal.pntd.0001512 (PMC3279362; doi:10.1371/journal.pntd.0001512)
Supplement: Table S2 — Homozygous SNPs found after resequencing the genome of L. major Friedlin. All the SNPs were identified in L. major Friedlin wild-type and in the mutants MF80.3 and MF80.5. The symbol * corresponds to a potential stop codon. (DOC) [file pntd.0001512.s006.doc]

### Table S2. Homozygous SNPs found after resequencing the genome of *L. major* Friedlin. All the SNPs were identified in *L. major* Friedlin wild-type and in the mutants MF80.3 and MF80.5.

| **Chromosome** | **Gene** | **Position** | **Position in the gene** | **Reference base in GeneDB** | **Base in *L. major* Friedlin and MF mutants** | **Amino acid position** | **Reference amino acid in GeneDB** | **Amino acid in *L. major* Friedlin and MF mutants** |
| --- | --- | --- | --- | --- | --- | --- | --- | --- |
| 1 | LmjF01.0250 | 65798 | 188 | A | G | 63 | L | P |
| 4 | LmjF04.1200 | 455810 | 355 | A | G | 119 | F | L |
| 9 | LmjF09.0140 | 62288 | 264 | T | C | 88 | R | R |
| 13 | LmjF13.1130 | 395697 | 1579 | C | T | 527 | V | I |
| 18 | LmjF18.0530 | 219450 | 1146 | T | C | 382 | W | W |
| 22 | LmjF22.1500 | 615011 | 1036 | T | C | 346 | S | P |
| 27 | LmjF27.1660 | 684019 | 1223 | T | C | 408 | V | A |
| 27 | LmjF27.1670 | 688250 | 361 | A | G | 121 | I | V |
| 27 | LmjF27.1670 | 688252 | 363 | C | T | 121 | I | I |
| 27 | LmjF27.1730 | 714540 | 12 | T | C | 4 | C | C |
| 28 | LmjF28.0410 | 134853 | 1834 | G | C | 612 | P | A |
| 30 | LmjF30.0100 | 31915 | 1993 | C | A | 665 | V | L |
| 30 | LmjF30.1280 | 442972 | 1749 | C | T | 583 | Y | Y |
| 30 | LmjF30.1620 | 582797 | 49 | T | C | 17 | F | L |
| 30 | LmjF30.1960 | 729367 | 1522 | T | G | 508 | * | E |
| 30 | LmjF30.2210 | 848927 | 3364 | A | G | 1122 | L | L |
| 34 | LmjF30.3430 | 1302509 | 232 | A | G | 78 | I | V |
| 34 | LmjF34.3070 | 1371482 | 963 | A | C | 321 | * | Y |
| 34 | LmjF34.3520 | 1531492 | 181 | T | G | 61 | C | G |
| 36 | LmjF36.0910 | 345725 | 4474 | T | C | 1492 | S | P |

The symbol * corresponds to a potential stop codon.
