# Supplementary material for: Multiple Mutations in Heterogeneous Miltefosine-Resistant Leishmania major Population as Determined by Whole Genome Sequencing
Source: PLoS Negl Trop Dis. 2012 Feb 14;6(2):e1512. doi: 10.1371/journal.pntd.0001512 (PMC3279362; doi:10.1371/journal.pntd.0001512)
Supplement: Table S3 — Heterozygous allelic polymorphisms in L. major Friedlin. All the same SNPs were identified in L. major Friedlin wild-type and in the mutants MF80.3 and MF80.5. (DOC) [file pntd.0001512.s007.doc]

### Table S3. Heterozygous allelic polymorphisms in *L. major* Friedlin. All the same SNPs were identified in *L. major* Friedlin wild-type and in the mutants MF80.3 and MF80.5.

| **Chromosome** | **Position** | **Reference base in GeneDB** | **Base as deduced from NGS** | ***L. major* gene ID** | **Position in the gene** | **Amino acid change** |
| --- | --- | --- | --- | --- | --- | --- |
| 3 | 264082 | A | A or G | LmjF03.0690 | 2715 | D905D |
| 3 | 275115 | C | C or T | LmjF03.0700 | 942 | E314E |
| 3 | 280590 | T | T or C | LmjF03.0720 | 280 | T94A |
| 3 | 280243 | G | G or A | LmjF03.0720 | 627 | F209E |
| 3 | 347038 | C | C or T | LmjF03.0860 | 1096 | S366G |
| 4 | 25245 | A | A or G | LmjF04.0050 | 1377 | A459A |
| 4 | 80398 | T | T or C | LmjF04.0240 | 518 | V173A |
| 8 | 73880 | G | G or A | LmjF08.0190 | 2116 | V706I |
| 8 | 454147 | G | G or A | LmjF08.1000 | 1811 | T604M |
| 8 | 454086 | G | G or A | LmjF08.1000 | 1872 | S624S |
| 8 | 449259 | G | G or A | LmjF08.1000 | 6699 | V2233V |
| 8 | 448974 | C | C or T | LmjF08.1000 | 6984 | A2328A |
| 8 | 480590 | C | C or T | LmjF08.1090 | 1215 | A405A |
| 8 | 514476 | C | C or T | LmjF08.1150 | 2003 | A668V |
| 8 | 514704 | T | T or C | LmjF08.1150 | 2231 | M744T |
| 9 | 566252 | A | A or G | LmjF09.1520 | 591 | V197V |
| 12 | 472090 | G | G or A | LmjF12.0760 | 574 | A192T |
| 12 | 547640 | A | A or C | LmjF12.1090 | 290 | E97A |
| 17 | 235920 | T | T or C | LmjF17.0470 | 1026 | T342T |
| 17 | 626085 | T | T or C | LmjF17.1315 | 857 | L286P |
| 17 | 672958 | G | G or A | LmjF17.1440 | 3676 | V1226I |
| 19 | 212339 | T | T or C | LmjF19.0560 | 1805 | M602T |
| 19 | 494355 | A | A or G | LmjF19.1140 | 1289 | S430N |
| 20 | 286000 | G | G or A | LmjF20.0705 | 342 | S114S |
| 20 | 487152 | G | G or A | LmjF20.1100 | 4929 | S1643S |
| 22 | 697546 | G | G or T | LmjF22.1690 | 2183 | G728V |
| 23 | 213988 | C | C or T | LmjF23.1590 | 788 | S263L |
| 24 | 271835 | T | T or G | LmjF24.0770 | 502 | S168A |
| 24 | 649385 | G | G or A | LmjF24.1730 | 1148 | P383L |
| 29 | 62836 | A | A or C | LmjF29.0190 | 2509 | L837V |
| 29 | 1063051 | T | T or G | LmjF29.2440 | 2149 | L717V |
| 31 | 1253265 | T | T or C | LmjF31.2570 | 3201 | R1067R |
| 32 | 1041134 | G | G or A | LmjF32.2650 | 3355 | L1119F |
| 32 | 1591898 | T | T or C | LmjF32.3970 | 109 | S37P |
| 34 | 505738 | T | T or C | LmjF34.1130 | 6960 | R2320R |
| 34 | 560255 | G | G or A | LmjF34.1240 | 920 | A307V |
| 34 | 1016135 | T | T or C | LmjF34.2260 | 156 | E52E |
| 35 | 2086387 | G | G or A | LmjF35.5390 | 17451 | R5817R |
| 36 | 1489286 | A | A or T | LmjF36.3900 | 669 | R223R |
| 36 | 1829384 | A | A or C | LmjF36.4720 | 1564 | R522S |
| 36 | 1893453 | T | T or C | LmjF36.4900 | 2280 | L760L |
| 36 | 1923316 | A | A or G | LmjF36.4970 | 544 | C182R |
